# Effect of Secondary Foaming on the Structural Properties of Polyurethane Polishing Pad

**DOI:** 10.3390/ma17112759

**Published:** 2024-06-05

**Authors:** Minxuan Chen, Zhenlin Jiang, Min Zhu, Baoxiu Wang, Jiapeng Chen, Wenjun Wang

**Affiliations:** 1 College of Chemistry and Chemical Engineering, Advanced Materials Research Center for Nano Manufacturing, Shanghai University of Engineering Science, Shanghai 201620, China; m340121118@sues.edu.cn (M.C.); zhu0304min@163.com (M.Z.); wangbaoxiu@sues.edu.cn (B.W.); 15937311032@163.com (J.C.); 04200004@sues.edu.cn (W.W.); 2 Key Laboratory of High Performance Fibers and Products, Donghua University, Shanghai 201620, China; 3 College of Chemical Engineering, Nanjing Tech University, Nanjing 211816, China

**Keywords:** polyurethane polishing pad, sodium hydrogen carbonate, ammonium bicarbonate, porosity

## Abstract

Polyurethane polishing pads are important in chemical mechanical polishing (CMP). Thus, understanding how to decrease the density but increase the porosity is a crucial aspect of improving the efficiency of a polyurethane polishing pad. According to the principle of gas generation by thermal decomposition of sodium bicarbonate and ammonium bicarbonate, polyurethane polishing pad was prepared by a secondary foaming method. The influence of adding such an inorganic foaming agent as an auxiliary foaming agent on the structure, physical properties, and mechanical properties of polyurethane polishing pads was discussed. The results showed that compared with the polyurethane polishing pad without an inorganic foaming agent, the open-pore structure increased, the density decreased, and the porosity and water absorption increased significantly. The highest porosity and material removal rate (MRR) with sodium bicarbonate added was 3.3% higher than those without sodium bicarbonate and 33.8% higher than those without sodium bicarbonate. In addition, the highest porosity and MRR with ammonium bicarbonate were 7.2% higher and 47.8% higher than those without ammonium bicarbonate. Therefore, it was finally concluded that the optimum amount of sodium bicarbonate to be added was 3 wt%, and the optimum amount of ammonium bicarbonate to be added was 1 wt%.

## 1. Introduction

Polyurethane polishing pads have been widely used in chemical mechanical polishing (CMP) [1] because of their excellent wear resistance, chemical stability [2], elasticity [3], and porous structure [4,5]. This stems from the phase separation structure of the soft and hard segments of the molecular chain [2,6]. The performance of polyurethane polishing pads is mainly determined by three factors: porosity, cellular microstructure and polymer properties [7]. Porosity is critical to the mechanical properties of polyurethane polishing pads to effectively remove the material during the CMP process [8]. Therefore, decreasing the density while increasing the porosity is the key to improving the efficiency of the polyurethane polishing pad. At present, nanoparticles have been introduced into the matrix in many studies [9,10] to improve the cell structure and promote bubble nucleation. Still, this method has the disadvantage of uneven dispersion of nanoparticles [11], which leads to irregular bubble hole structure and a significant reduction in foaming efficiency.

Secondary foaming refers to a process in which material is made to foam again under specific conditions to increase its foaming rate or change its physical properties. Aimin Zhang et al. [12] provided an efficient method for preparing low-density poly (ether block amide) (PEBA) foams without increasing energy consumption. Firstly, PEBA foam with controllable high density was prepared by microcellular foaming at a relatively low foaming temperature. Afterward, high-density pre-foaming PEBA samples were further foamed into low-density pre-foaming samples by secondary microcellular foaming at a relatively high foaming temperature. Under the same foaming parameters, compared with the traditional microcellular foaming process, the pre-foaming process can not only shorten the saturation time in the foaming process, but also help PEBA achieve a higher expansion ratio. Fuquan Deng et al. [13] used a double-foaming system of 4,4′-oxydibenzenesulfonyl hydrazide (OBSH) and azodicarbonamide (AC) to construct a double-peak unit structure. This method could form pores of different sizes at the same temperature, enhance the tear strength of the foam, and improve the crystallization characteristics. This study shows that the foam density can be effectively decreased by changing the foaming process and adopting the secondary foaming method. Still, this method is rarely used for polyurethane foaming at present. The commonly used polyurethane foaming one-step method produces foams with high density and low porosity, which are not easy to form, alongside other issues; a secondary foaming method can be used in the preparation of polyurethane polishing pads to make up for the shortcomings of the one-step method to improve polishing efficiency and polishing quality.

A foaming agent is a key additive in releasing the gas required for foaming polyurethane polishing pads [14], and the type and dosage of the blowing agent affect the density and other properties of the polyurethane polishing pad [15,16]. Hence, the choice of blowing agent is particularly important [17]. Compared with physical blowing agents, chemical blowing agents have obvious advantages in compressive strength [18], environmental performance [17], thermal insulation effect [19], bonding performance [20], and bubble structure [21]. Mohamad Saed Hussein et al. [22] discussed the effect of sodium bicarbonate as a blowing agent in epoxy resin. It was found that the microstructure of the epoxy system changed from dense phase to porous phase with the increase in blowing agent content from 0 phr to 20 phr. With the increase in sodium bicarbonate content, the density decreased, but the viscosity increased slightly. When forming an epoxy foam, the more sodium bicarbonate content, the lower the bending strength, bending modulus and fracture toughness. T. Fawzi et al. [23] added water and sodium bicarbonate as blowing agents to the PU mixture at the same time. A permeable open-pore structure was formed, and FT-IR analysis showed that these blowing agents had no effect on the performance of the polyurethane foam. In contrast, sodium bicarbonate as a blowing agent produces a high density of closed-vesicle pore samples. This morphology may affect thermal dispersion, which in turn leads to the formation of hard segments and an increase in mechanical properties, illustrating that sodium bicarbonate can be applied to improve tear resistance. Currently, most studies use sodium bicarbonate as a foaming agent to study its effect on materials, while ammonium bicarbonate, also a green, inexpensive, heat-decomposable inorganic foaming agent [24], is seldom used in foaming. Therefore, the addition of inorganic foaming agents such as sodium bicarbonate and ammonium bicarbonate can be used to improve the porosity and mechanical properties of polyurethane polishing pads [25]. However, no studies have been conducted to combine inorganic blowing agents with secondary foaming methods to change the porosity of polyurethane polishing pads.

Aiming at the existing one-step polyurethane foam preparation process, with problems such as a low product polishing efficiency, we need to prepare polyurethane polishing pads with low density and large porosity to improve the polishing efficiency and reduce the production time. This study employed the secondary foaming method to first perform a water reaction with isocyanic acid to produce gas after high-speed stirring. Subsequently, an inorganic foaming agent (sodium bicarbonate and ammonium bicarbonate) was prepared to generate gas-assisted foaming after heat decomposition to prepare a polyurethane polishing pad and explore its properties. Hence, a new idea is provided for material screening and process optimization of polyurethane polishing pads.

## 2. Materials and Methods

### 2.1. Raw Materials

Polypropylene glycol PPG1000 was obtained from Jiangsu Hai’an Petrochemical Plant (Hai’an, China). CASE Polyether polyol D204 was purchased from Jiahua Chemical Co., Ltd. (Shanghai, China). Isophorone diisocyanate (IPDI) was provided by Shanghai Taitan Technology Co., Ltd. (Shanghai, China). Diphenylmethane diisocyanate (Polymerization MDI 44V20L) was acquired from Jining Ribuluo Biological Technology Co., Ltd. (Jining, China). Dibutyltin dilaurate (DBTDL) and sodium hydrogen carbonate (NaHCO_3_) was provided by Shanghai Taitan Technology Co., Ltd. (Shanghai, China). Ammonium bicarbonate (NH_4_CO_3_) was purchased from Wuxi Zhanwang Chemical Reagent Co., Ltd. (Wuxi, China).

### 2.2. Preparation of Sample

#### 2.2.1. Preparation of Polyurethane Prepolymers

An amount of 57 wt% of the weighed PPG1000 was added into a three-mouth flask and dehydrated in a vacuum at 120 °C in an oil bath for 1 h and then naturally cooled down to about 80 °C. Afterwards, 38 wt% IPDI was added to PPG1000, stirred evenly at 80 °C. DBTDL, as a catalyst, was added with a ratio in the whole prepolymer system of 5 wt% and then reacted at 80 °C for 2 h. The whole reaction process was carried out under the protection of N_2_. NCO content in the prepolymer was determined by using the di-n-butylamine toluene method. When the content reached the preset theoretical value (10.08%), the reaction was stopped, and the prepolymer was vacuumed and sealed.

#### 2.2.2. Preparation of Polyurethane Polishing Pad

The prepared prepolymer was poured into a plastic box. An amount of 55.37 wt% polyether polyol D204, 0.17 wt% of blowing agent water, 0.17 wt% DBTDL, and different quantities of NaHCO_3_ (1 wt%, 2 wt%, 3 wt%, 5 wt%) and NH_4_CO_3_ (0.5 wt%, 1 wt%, 2 wt%, 3 wt%, 5 wt%) were mixed and stirred well, and then 44.29 wt% polymerized MDI was added. A mechanical stirring device was utilized to stir at a high speed of 2000 r/min. The mixture was poured into the mold and stood at room temperature for 40 min. Finally, the prepared polishing pad after curing was taken out and placed in an oven for secondary-foaming curing for 24 h. 

### 2.3. Test and Characterization

1. Pyrolysis test: The decomposition temperatures of sodium bicarbonate and ammonium bicarbonate were determined using synchronous thermal analysis TG-DSC (STA 449 F3, German Netzsch Company, Selb, Germany). The test range was 30–200 °C, and the temperature increase rate was 10 °C/min.
2. Fourier infrared spectroscopy test: Samples were tested with Fourier transform infrared spectrometer (Type 8700, American Nicolet Company, Madison, WI, USA). The scanning wavelength range of the instrument was 4000~400 cm^−1^, and air was selected as the background.
3. Observation of bubble structure: The sample was cut into thin slices and dried in an oven for 24 h. The surface of the sample was treated with uniform gold spraying. The morphology of the sample surface was obtained by scanning electron microscopy (GeminiSEM 300, German ZEISS Company, Oberkochen, Germany).
4. Aperture distribution test: The sample was cut into 10 mm × 10 mm × 10 mm cubes with a mass of 0.1–0.4 g and dried. The pore diameter of the sample was tested using a mercury injection instrument (PoreMaster60, Austria Anton Paar Company, Graz, Austria).
5. Density measurement: The sample was cut into 30 mm × 30 mm × 30 mm cubes, and the length, width, and height of the sample were measured and averaged from three different positions. The mass and volume were measured, and the density was calculated by Equation (1). Each group of samples was measured 5 times and averaged.

(1) $$\rho =\frac{m}{v}\times 10^{3}$$

where ρ is apparent density (g/cm^3^), m is the sample mass (g), and v represents the sample volume (mm^3^)

6. Measurement of volumetric water absorption: The sample was cut into 100 mm × 100 mm × 20 mm cubes with a weight of *m*_1_. The sample was immersed in a container about 20 mm away from the liquid level. After 96 h, the water on the surface was removed and weighed, *m*_2_.

(2) $$W=\frac{m_{2}-m_{1}}{m_{1}}\times 100\%$$

(3) Wv=W×ρ

where W denotes specific absorption of quality (%), $m_{1}$ is mass before water absorption (g), $m_{2}$ is mass after water absorption (g), Wv is water absorption by volume (%), and ρ is density of the sample under natural drying (g/cm^3^).

7. Porosity test: Porosity referred to the percentage of the pore volume inside the foam to the total foam volume and was determined by the density of the foam and the matrix density of the polymer.

(4) $$P=1-\frac{\rho }{\rho_{0}}$$

where P represents porosity (%), ρ is density of sample, and $\rho_{0}$ is density of the corresponding foam material base (polyurethane = 1200 kg/m^3^).

8. Hardness determination: The Shore C hardness of the sample was measured. Five points were calculated for each group of samples and averaged.
9. Compression strength test: The sample was made into a cube of 30 mm × 30 mm × 30 mm, and the compression mode of the material universal testing machine (AGS-X, Japan Shimadzu Company, Kyoto, Japan) was used to compress the sample by 6 mm at a compression speed of 3 mm/min and a compression rate of 20%. For the compression Poisson ratio test, the sample was made into a cube of 30 mm × 30 mm × 30 mm, a straight line was drawn perpendicular to the direction of compression on the sample as a test point, and then vernier calipers were used to measure the original length of the sample, the original thickness, and its dimensional changes under different strains; then, using the Poisson ratio formula, the foam Poisson ratio was calculated. An average value was taken after three measurements.

$$\nu =\frac{\varepsilon_{x}}{\varepsilon_{y}}=\frac{∆l_{x}}{\Delta l_{y}}\cdot \frac{l_{y}}{l_{x}}$$

where ν Poisson’s ratio, $\varepsilon_{x}$ is the transverse strain (%), $\varepsilon_{y}$ is the longitudinal strain (%), $∆l_{x}$ is the lateral length change (mm), $\Delta l_{y}$ is the longitudinal length change(mm), $l_{x}$ is the original length of the horizontal (mm), and $l_{y}$ is the original length in the longitudinal direction (mm).

10. Test of polishing performance: Polishing performance was tested on the SK-380A polishing machine. The polishing workpiece was K9 glass with a diameter of 100 mm and a thickness of 0.5 mm. The concentration of silicon dioxide (130 nm), polishing fluid flow rate, polishing head, polishing disc, and polishing time were, respectively, set at 20%, 30 mL/min, 50 rpm, 50 rpm, and 20 min. The material removal rate (MRR) was calculated.

(5) $$MRR=\frac{∆m\times 10^{7}}{\rho \pi r^{2}t}\;$$

where MRR material removal rate (nm/min), ∆m is the quality difference of the wafer before and after polishing (g), ρ is density of K9 glass (ρ = 2.2 g/cm^3^), r is wafer radius (cm), and t indicates polishing time (min).

## 3. Result and Discussion

### 3.1. Thermal Decomposition Analysis

**Figure 1:**
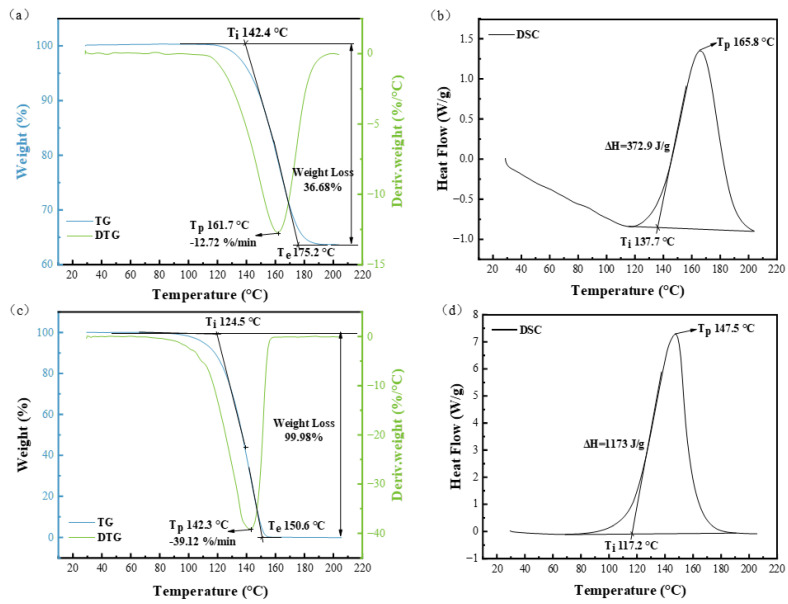
Thermal decomposition diagrams of NaHCO_3_ and NH_4_HCO_3_: TG−DTG curve (**a**) and DSC curve (**b**) of NaHCO_3_, and TG−DTG curve (**c**) and DSC curve (**d**) of NH_4_HCO_3_.

The thermal decomposition behaviors of NaHCO_3_ and NH_4_HCO_3_ were analyzed, as shown in Figure 1. According to the DSC curve, the NaHCO_3_ sample began to undergo exothermic decomposition reaction at 137.7 °C, while the TG curve began to lose weight at 142.4 °C (reaching the highest rate at 161.7 °C). When the temperature reached about 175.2 °C, the DSC curve and TG curve ended almost simultaneously. The weight loss of the sample was 36.88% (CO_2_ and H_2_O), and the remaining sample composition was presumed to be sodium carbonate. According to the DSC curve, the NH_4_HCO_3_ sample began to undergo an exothermic decomposition reaction at 117.2 °C, while the TG curve began to lose weight at 124.5 °C, with the highest rate at 142.3 °C. At 150.6 °C, the DSC curve and TG curve ended almost at the same time, and the sample was completely decomposed (ammonium bicarbonate was decomposed into CO_2_, NH_3_, and H_2_O). Therefore, the temperatures chosen for secondary foaming in the preparation of polyurethane polishing pads were 150 °C for NaHCO_3_ and 140 °C for NH_4_HCO_3_.

### 3.2. Structural Characterization

**Figure 2:**
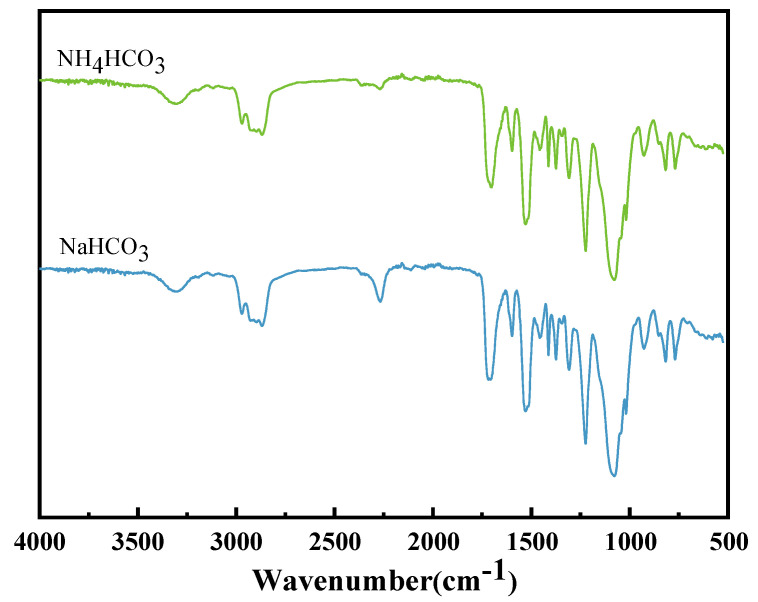
FT−IR spectrum of polyurethane foam with added NaHCO_3_ and NH_4_HCO_3_.

The sample with 1 wt% of NaHCO_3_ and NH_4_HCO_3_ was tested by infrared (Figure 2).

According to Figure 2, for the NaHCO_3_ curve, the absorption peak at 3311 cm^−1^ is the stretching vibration of hydroxyl O–H, and the absorption peak at 3000–2800 cm^−1^ corresponds to the stretching vibration of alkyl C–H. The absorption peaks at 2267 cm^−1^ and 1717 cm^−1^ are attributed to the antisymmetric stretching vibration of isocyanate N=C=O and the stretching vibration of isocyanate C=O [26]. Additionally, the absorption peaks at 1596, 1529, and 1412 cm^−1^ are related to the skeleton vibration of the benzene ring, and the absorption peak at 1456 cm^−1^ is the variable angle vibration of methylene. The absorption peaks at 1456 and 1373 cm^−1^ are the antisymmetric and symmetric variable angle vibration of methyl. The adsorption peaks at 1307 cm^−1^, 1223 cm^−1^, and 1077 cm^−1^ are associated with the distortion vibration of the methylene group, the stretching vibration of ester C-O [27], and the stretching vibration of ether C–O–C [28]. The absorption peak at 1017 cm^−1^ is the in-plane bending vibration of benzene ring C–H, the oscillating vibration of methyl group is 926 cm^−1^, and the out-of-plane bending vibration of benzene ring C–H are at 816 and 758 cm^−1^. Theoretically, no strong absorption peak appears near 1654 and 688 cm^−1^ for NaHCO_3_ [29], suggesting that sodium bicarbonate has been completely decomposed. The NH_4_CO_3_ atlas is basically the same, and the absorption peak at 2363 cm^−1^ is the antisymmetric stretching vibration of CO_2_ in the air. No strong absorption peaks at 1280, 1600, 830, or 700 cm^−1^ were observed for NH_4_HCO_3_ [30]. This indicates that NH_4_HCO_3_ was completely decomposed.

**Figure 3:**
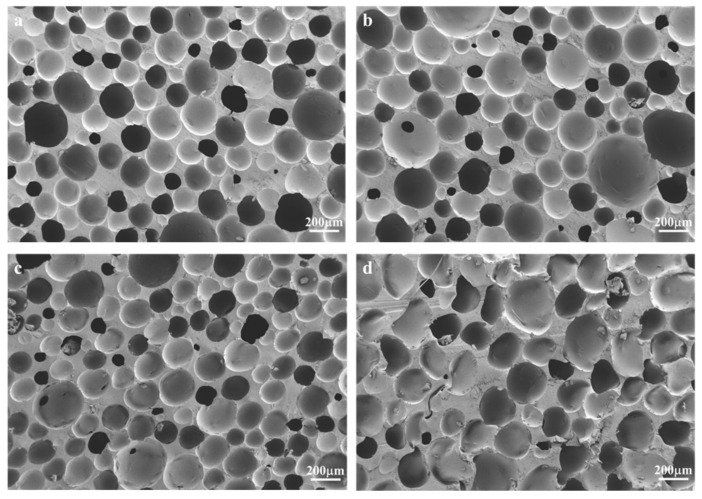
SEM images of foamed samples with added NaHCO_3_ ((**a**) 1 wt%, (**b**) 2 wt%, (**c**) 3 wt%, (**d**) 5 wt%).

According to Figure 3, the open pore structure of the sample gradually increases with the increase in the amount of NaHCO_3_.

When adding 1 wt% of NaHCO_3_, the pore size is relatively regular and circular. With the increase in the addition amount, the open-pore structure gradually begins to appear, and the pore aperture gradually becomes larger [31] but still maintains a relatively regular circular shape. When 3 wt% of NaHCO_3_ was added, the open-pore structure increased, and the pore size gradually changed from circular to oval. When the amount of NaHCO_3_ increased to 5 wt%, the pore structure became irregular, the pore wall thickness increased, and pore collapse occurred.

**Figure 4:**
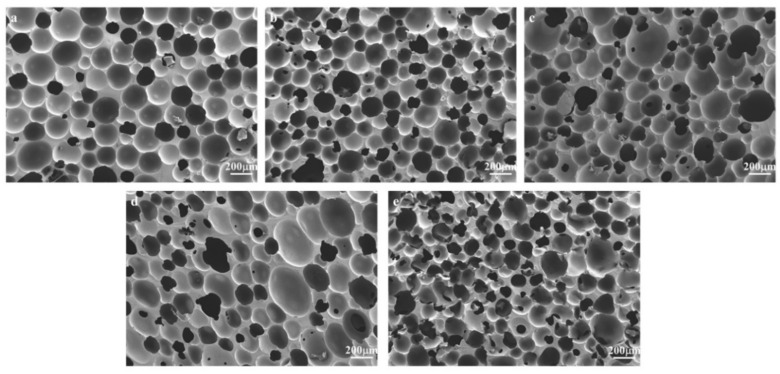
SEM images of foamed samples with added NH_4_CO_3_ ((**a**) 0.5 wt%, (**b**) 1 wt%, (**c**) 2 wt%, (**d**) 3 wt%, (**e**) 5 wt%).

As can be seen in Figure 4, at an NH_4_CO_3_ addition of 0.5 wt%, the bubble pores have a circular structure.

Additionally, there are many obvious structures that were opened in the wall of the bubble pores. This can be attributed to the fact that carbon dioxide in the pores cannot form enough pressure to break through the pore walls to form open holes. With the addition of 1 wt%, many open-pore structures appear, and most of the bubble pores are relatively uniformly round. When the addition amount of NH_4_CO_3_ gradually increased to 2 wt%, although there were still open pores, the shape of the cell began to become uneven, the distribution was irregular, and the pore diameter became larger. When adding 3 wt%, the bubble pore became elliptical, there was a merged bubble behavior between the small bubbles, and some of the bubble pores burst. At an additional amount of NH_4_CO_3_ of 5 wt%, a large number of cell holes collapsed, and the shape was very irregular.

**Figure 5:**
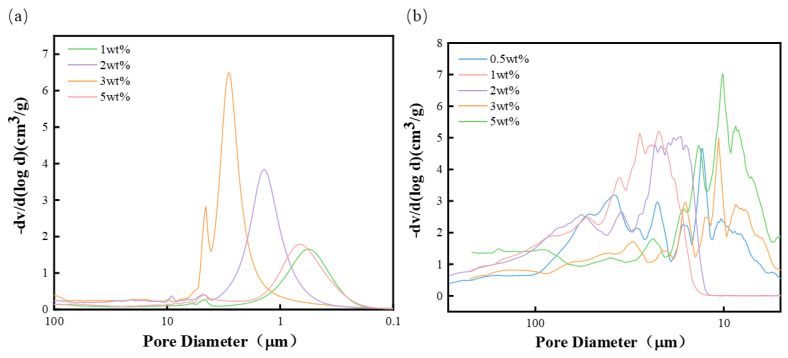
Aperture distribution curves of blowing agents with different additive amounts: (**a**) NaHCO_3_ and (**b**) NH_4_CO_3_.

Mercury injection test was carried out on the foam sample. As shown in Figure 5, the main peak addition amount of the foam sample aperture prepared by adding NaHCO_3_ was between 1 and 5 μm when the addition amount was 2 wt% and 3 wt%, and between 0.3 and 1 μm when the addition amount was 1 wt% and 5 wt%.

The main peak of the aperture of the foam sample prepared by adding NH_4_CO_3_ mainly ranges from 9 to 37 μm. This indicates that the aperture distribution of the prepared polyurethane foam sample is relatively concentrated, mainly with large pores. Moreover, the pore size of the sample prepared by NH_4_CO_3_ is basically larger than that prepared by NaHCO_3_. This phenomenon indirectly verifies why the performance data, such as volume water absorption of NH_4_CO_3_ in subsequent tests, are higher than those of NaHCO_3_.

### 3.3. Physical Performance

**Figure 6:**
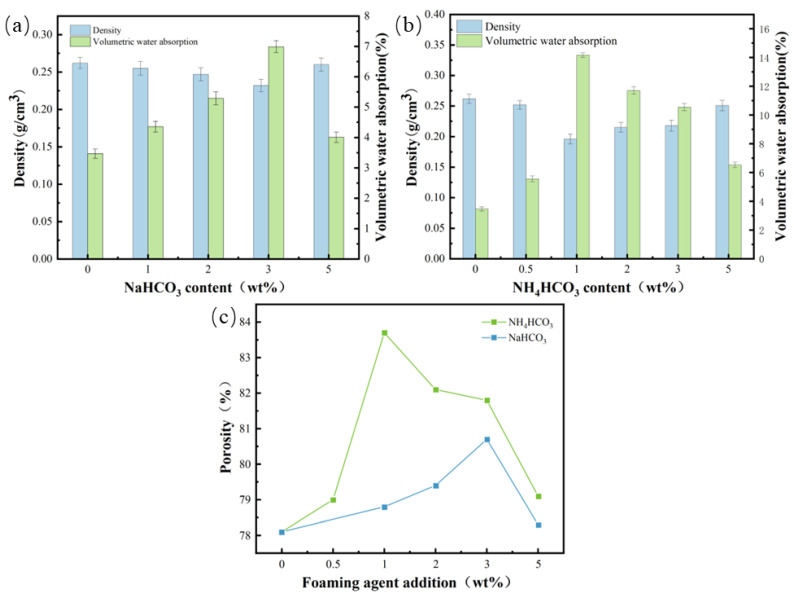
Physical properties of blowing agents with different additions (**a**) density volume water absorption of samples with added NaHCO_3_, (**b**) density volume water absorption of samples with added NH_4_CO_3_, and (**c**) porosity.

As can be seen from Figure 6, with the addition of inorganic blowing agent, the density of the material will decrease, and the volume water absorption and porosity will increase.

The density of the sample without the addition of sodium bicarbonate and ammonium bicarbonate is 0.262 g/cm^3^, the volume water absorption is 3.47%, and the porosity is 78.1%, while after the addition of sodium bicarbonate and ammonium bicarbonate, it can be seen in Figure 6 that the density of the sample decreases, and the volume water absorption and the porosity increase. The reduction in weight is achieved through the generation of gas, which is obtained thermally by the decomposition of sodium bicarbonate and ammonium bicarbonate, respectively.

When the dosage of NaHCO_3_ as a blowing agent was 3 wt%, the minimum density was 0.232 g/cm^3^, the volume water absorption was 6.99%, and the porosity was 80.7%. When adding 1 wt% of NH_4_CO_3_ as the blowing agent, the minimum density, the volume water absorption rate, and the porosity, respectively, reached 0.196 g/cm^3^, 14.17%, and 83.7%. After adding the blowing agent, the blowing agent produced bubbles inside the system, forming a bubble-cell structure. As a result, the porosity of the material increased while the density decreased [32]. The density of the material before the addition of 5 wt% correlated with the increase and decrease in the addition of NaHCO_3_ and NH_4_HCO_3_; this is because NaHCO_3_ and NH_4_HCO_3_, due to heat, began to decompose, producing carbon dioxide, which formed bubbles inside the material. With the increase in the addition of NaHCO_3_ and NH_4_HCO_3_, there was a gradual production of more and more gas, and the formed foam pore size gradually increased. The pore size of the foam formed gradually increased, and the gas broke through the wall of the bubble holes to form an open-pore structure, which made the material structure looser, and the density decreased. When the additional amount reached 5 wt% or even more, the gas produced inside the material was too much to cause the foaming reaction to continue; the polyurethane could not dissolve the excess gas, causing excessive pressure in the system, so that the foam was produced by the constant contact and extrusion of small bubbles, and the surrounding bubbles quickly formed large bubbles that overflowed, resulting in the collapse of the bubble holes, reducing the porosity of the material. In addition, the reaction rate was too fast, so the gel time and foaming time were not equal, which is required for foam stability, and so the stability of the material was relatively loose and the density was reduced [33]. This meant the bubble holes were easily ruptured, and the regular and complete structure of the bubble holes was reduced, resulting in an increase in the density of the material.

The chemical equations for the thermal decomposition of sodium bicarbonate and ammonium bicarbonate are as follows:

$$2{NaHCO}_{3}≜{NaCO}_{3}+{CO}_{2}↑+\;H_{2}O$$

$${NH}_{4}{HCO}_{3}≜{NH}_{3}↑+H_{2}O+{CO}_{2}↑$$

From the chemical equation, it can be seen that the amount of gas produced by the reaction of ammonium bicarbonate is greater than that of sodium bicarbonate at the same molar ratio, and the heating temperature of ammonium bicarbonate [34] is lower than that of sodium bicarbonate [35]. The gassing amount of ammonium bicarbonate is about 850 mL/g, and the gassing amount of sodium bicarbonate is about 267 mL/g, and the carbon dioxide produced by the thermal decomposition of sodium bicarbonate is only half of the theoretical amount. Therefore, with the same amount of addition, more gas will be generated inside the foam material with ammonium bicarbonate, so as to obtain a larger porosity.

### 3.4. Mechanical Performance

**Figure 7:**
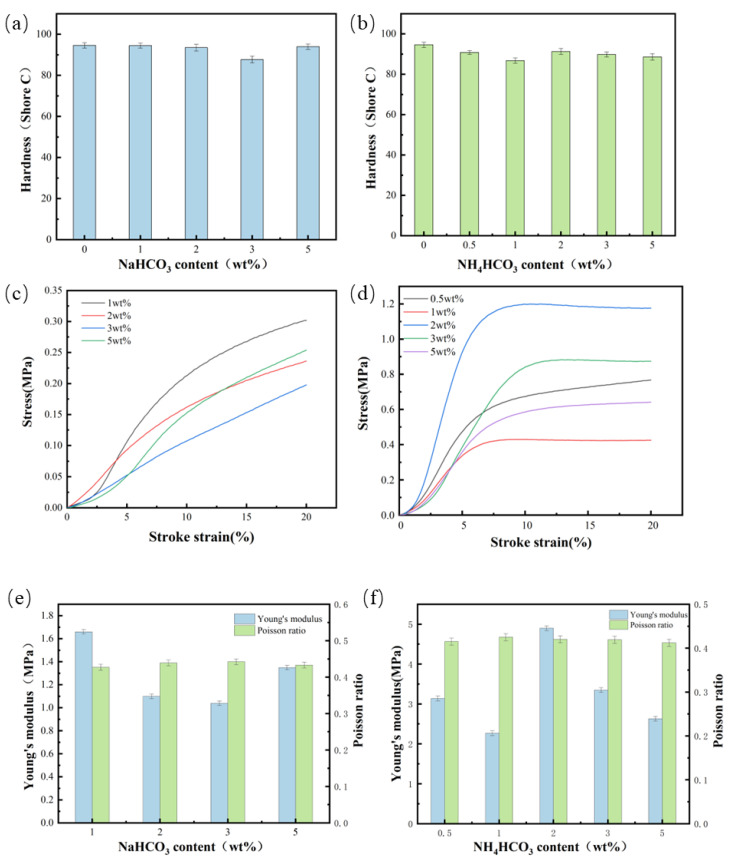
Mechanical properties of blowing agents with different additions (**a**) hardness of samples with added NaHCO_3_, (**b**) hardness of samples with added NH_4_CO_3_, (**c**) compressive strength curve of samples with added NaHCO_3_, and (**d**) compressive strength curve of samples with added NH_4_CO_3_, (**e**) Young’s modulus and Poisson’s ratio with the addition of sodium bicarbonate, (**f**) Young’s modulus and Poisson’s ratio with added ammonium bicarbonate.

As Figure 7 shows, the polyurethane polishing pad must have a certain surface rigidity to ensure a high grinding ability.

It can be found that the average hardness of the polyurethane polishing pad made by NaHCO_3_ as a blowing agent or NH_4_CO_3_ is about Shore C 90. This hardness range ensures that the polishing pad can provide sufficient support to the polishing fluid and the workpiece during the polishing process. When NaHCO_3_ was used as the blowing agent, the compressive strength decreased with the increase in the amount of the blowing agent and then tended to be stable. When using NH_4_CO_3_ as a blowing agent, the compressive strength first decreases and then increases, and finally tends to be stable. This is because the increase in the amount of foaming agent added increases the foaming rate of the material and increases the number of bubble holes. This decreases the force entity of the composite material, and the wall of some bubble holes becomes thinner or has gaps. Therefore, when the material is subjected to pressure, the phenomenon of stress concentration is prone to occur at the bubble hole. This becomes the stress failure point and is manifested in the reduction in compressive strength on a macroscopic level. When the density of the material increases, the compression strength will increase accordingly [23]. However, when adding more foaming agents of about 5 wt%, the air volume is too large, resulting in bubble collapse, or the number of parallel holes increases. The increase in the number of bubble holes decreases, but the pressure capacity decreases. Ultimately, there would be a decrease and a flat variation tendency. According to Figure 7e,f, the change rule of Young’s modulus and Poisson’s ratio of the material and the compression curve is basically the same: the Young’s modulus of the sample will increase with the increase in compression strength, and the larger the Young’s modulus, the better the resistance of the material to deformation, but its density will also increase accordingly, resulting in a decrease in porosity [36]; the Poisson’s ratio of the sample and the compression strength of the sample are inversely proportional, and a larger Poisson’s ratio indicates that the material has a larger volume change when subjected to force, which leads to the deformation of the molecular structure, resulting in a reduction in strength, so the material with a high Poisson’s ratio has a smaller density and a larger porosity [37].

### 3.5. Material Removal Rate

**Figure 8:**
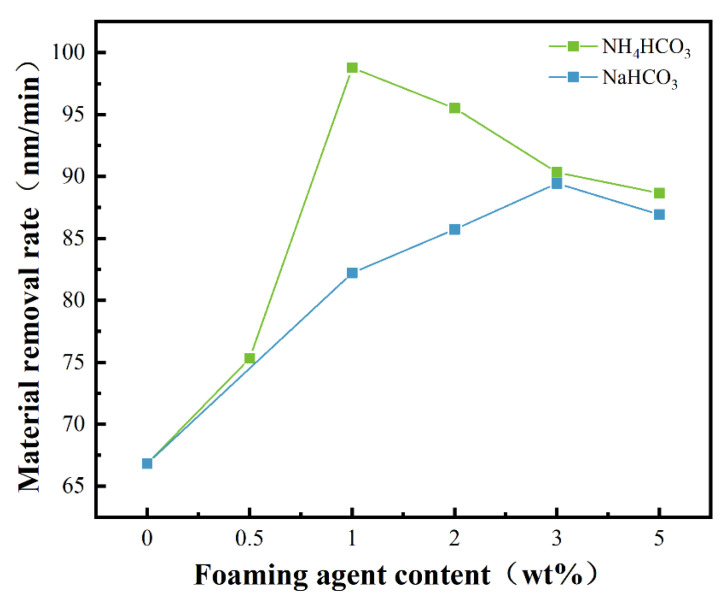
Material removal rate of polyurethane polishing pad.

The material removal rate (MRR) of the two groups of polyurethane polishing pads was tested and measured. As shown in Figure 8, when NaHCO_3_ was used as a blowing agent, the MRR first increased and then decreased with the increase in blowing agent addition.

The polishing pads with NaHCO_3_ and NH_4_HCO_3_ added were compared to the unadded MRR, and it was found that the highest MRR with NaHCO_3_ added was 33.8% higher than those without NaHCO_3_, and the highest MRR with NH_4_HCO_3_ added was 47.8% higher than those without NH_4_HCO_3_. This result proves that the addition of such inorganic foaming agents can improve the polishing efficiency of polishing pads.

The maximum MRR was 89.45 nm/min when the addition amount was 3 wt%. When using NH_4_CO_3_ as a blowing agent, the MRR first increased and then decreased with the increase in the amount of blowing agent, and the maximum MRR was 98.78 nm/min when the amount was 1 wt%. This can be explained by the fact that the porosity of the polishing pad gradually increases, and the storage capacity of the polishing liquid increases, so the contact area between the polishing liquid and the workpiece increases and the oxidation rate of the workpiece surface is accelerated with a higher material removal rate. Moreover, the greater the hardness of the polishing pad in the polishing process, the greater the bearing capacity and the more difficult it is for the foam to be deformed [38], so as to obtain a flatter workpiece surface. Then, it gradually decreases because the amount of blowing agent added is too much, and the bubble hole breaks. Meanwhile, the ability to transport impurities and polishing liquid is weakened, and the phenomenon of uneven force on the surface of the workpiece may occur. Therefore, there is a decrease in MRR.

## 4. Conclusions

This study mainly investigated the effect of secondary foaming on the structural properties of polyurethane polishing pads, using two inorganic blowing agents, NaHCO_3_ and NH_4_HCO_3_, compared with polishing pads without added inorganic blowing agents, and concluded that the optimal additive ratio of NaHCO_3_ is 3 wt%, and that of NH_4_HCO_3_ is 1 wt%. The pores of the vesicles in this ratio have a more open pore structure, a more uniform size, a larger porosity, and the highest MRR. The specific conclusions are as follows:

1. The polyurethane foam with NH_4_CO_3_ has a more open pore structure and larger aperture than that with NaHCO_3_. The open pore structure increases with the increase in foaming agent added, but adding too much will cause bubble collapse and lead to the deterioration of various properties of polyurethane foam polishing pad.
2. The polyurethane foam polishing pad added with NaHCO_3_ and NH_4_CO_3_ has a change in physical properties compared with the non-added polishing pad, the relative density decreases, and the porosity and volume water absorption increase significantly. With the increase in the addition of NaHCO_3_ and NH_4_CO_3_, the density will first decrease and then increase due to factors such as pore rupture, and the porosity and volume water absorption will first increase and then decrease.
3. The hardness of the two groups of polyurethane foam polishing pads added with NaHCO_3_ and NH_4_CO_3_ can be maintained at about 90 Shore C, meeting the basic hardness required for the polishing process. The appropriate addition of NaHCO_3_ and NH_4_CO_3_ can enhance the mechanical properties of the polyurethane foam polishing pad. Nevertheless, excessive dosage will decrease the mechanical properties of the material.
4. Compared with the polishing pad without the auxiliary foaming of NaHCO_3_ and NH_4_CO_3_, the material removal rate of the polishing pad prepared by the secondary foaming method was significantly improved, and the highest MRRs of NaHCO_3_ and NH_4_CO_3_ were 89.45 nm/min and 98.78 nm/min, respectively. Increasing the porosity while keeping the hardness basically unchanged can improve the MRR.

## Data Availability

The raw data supporting the conclusions of this article will be made available by the authors on request.
